# Surgical Diseases of Women—Ovarian Tumor, with Report of Cases

**Published:** 1864-09

**Authors:** J. F. Miner


### ART. III.—*Surgical Diseases of Women—Ovarian Tumor, with Report of Cases.* By J. F. MINER, M. D.

Cystic disease of the ovary has been for a long period recognized, and has of late years received cureful study by the most eminent and accurate observers, though in many respects it is still imperfectly understood, and great variety of opinions are entertained concerning almost all the essential conditions of its origin and development as well as the proper manner of its control or removal.

It is not proposed to discuss at any length the numerous questions which have been, and still are, agitated concerning the development or structure of the ovarian cyst, or the course or symptoms of the disease. Cystic degeneration of glandular structure is one of the most common forms of morbid action, and is occasionally observed in all or nearly all the larger glands; the testicle and ovary seem most often the seat of such disease, the kidneys and liver have been known to become similarly affected; this will be seen by one of the cases which will be hereafter related.

We suspect that there is present ovarian disease, upon the appearance of a slowly formed and gradually increasing abdominal tumor, at first situated distinctly upon one side, having a dull percussion sound, with the tympanitic sound of the intestines upon the opposite side, and at length a distinct fluctuation, with absence of the evidences of other conditions or diseases—pregnancy, ascites, or uterine tumor—and without any notable impairment of the general health or change in the common functions of life. The diagnosis is made more certain when the tumor is movable; if composed of several cysts the character is often obscured by this circumstance; fluctuation is thus rendered indistinct, and the location of the tumor cannot be so accurately determined. It should be constantly borne in mind that there are occasionally developed in the peritoneum or abdominal walls, or very rarely in the kidney, liver, or omentum, cystic tumors which are distinguishable with difficulty, or even not at all from ovarian cysts. Those from the liver or kidney are very liable to be confounded with them. The following case substantiates this latter statement:

Mrs. J. B. had for several years suffered from tumor in ovarian region; the general health was considerably impaired, but the growth of the tumor was but gradual, and what was quite remarkable, it would appear much smaller at times as if in process of disappearance. She had consulted many of the most distinguished physicians, and had become from the harmony of opinion expressed, satisfied of the presence of ovarian tumor. While making ready for church she suddenly exclaimed, "O! my head," and fell upon the floor, expiring in a few minutes. *Post mortem* examination was made by the writer, and four ounces of blood was found effused upon the right side of the brain; the tumor which had been regarded as undoubtedly ovarian by the most distinguished physicians in Boston and New York, proved upon examination to be cystic degeneration of the kidney. The morbid specimen weighed four pounds three ounces, and consisted of strong transparent fibrous sacs, the larger ones filled with serous fluid, while some of the smaller ones contained a thicker, more gelatinous material. The natural structure of the kidney was wholly absent.

We may sometimes almost complete our diagnosis by the introduction of exploring trocar; this is thought to be capable of demonstrating the nature of such disease, but this is also liable to mislead. All the morbid growths attached to any of the abdominal organs are liable to be attended by effusion of serum into the abdominal cavity, so that the mere fact of drawing off serous fluid would show nothing positively. A case which has been under my care for several years—a standing clinical case, and repeatedly tapped at the General Hospital, considered as thoroughly understood while living proved after death to be quite unlike what had ever been suspected. Differences of opinion had been expressed upon occasions of clinical presentation, and while one had supposed that the tumor was multilocular, and the small cysts contained within the larger, thus allowing twenty quarts of serum to escape, and leaving distinct round cysts unemptied, another had regarded the case as complicated with effusion into abdominal cavity, the tumors only becoming distinct after removal of effusion. The tumor proved to be ovarian, with cysts whose walls were so thick and unyielding that when evacuated the form was the same, and thus distension of the abdomen as great as before—the tumor was almost solid, the greater part being made up of fleshy material. The large quantity of serum was from the cavity of the abdomen.

When probable ovarian disease is present, what shall we advise? The difficulties and uncertainties of diagnosis are well understood. The absolute impossibility of knowing the exact condition is to be considered in arriving at proper reply to the question.

There is no doubt that tumors attached to the ovary are successfully removed; that in favorable cases the operation would be attended by favorable results in large proportion of cases, while at the same time it is not to be denied that patients live for years, and complete the residue of life with considerable comfort, suffering constantly of course from apprehension of impending danger, and occasionally being obliged to have the distension relieved by evacuation of the contents of the sac or sacs.

Mrs. R. of this city has now had appearances which almost unmistakably establish the fact of ovarian disease for about four years. During this period she has been tapped at two different times with evacuation of about ten quarts of serum upon each occasion. The re-accumulation is so slow that a year or more is required before distension produces great discomfort. When the fluid is all evacuated the evidences of disease are almost absent. The constitutional disturbance is very slight, and the tumor can with great difficulty, if at all, be distinguished.

She is fifty-six years old and pursuing a lady's ordinary round of pleasure and business. In this case I have never advised operation, though it is a case as likely to terminate favorably as any. The patient has been faithfully instructed into all the arguments for and against, and has thus far perhaps wisely preferred to submit to her present inconveniences and disabilities rather than accept the uncertainties attendant upon effort for permanent relief. Operation for radical cure is attended by great risk under all circumstances; the cavity of the abdomen cannot be opened without endangering life.

Other cases, not widely different, in many respects would be regarded as more urgently demanding operation.

Age is of great importance in determining the propriety of operation. The development of such disease at fifty, of slow growth, with little constitutional disturbance, would be considered very unlike similar condition at thirty or even forty years of age, though at all ages it is of serious import and liable to be attended by aggravated symptoms and unfavorable termina tion.

There are cases frequently presented which demand operation, and in which delay is dangerous and fatal. Mrs. B. of Buffalo, aged fifty-four, had suffered from ovarian tumor ten years, perhaps some longer than this. For the first five there was little inconvenience other than the weight, but the growth gradually increased and at the end of this term had become exceedingly large. Respiration was difficult and pain of existence so great, that death would be a pleasure compared with a life of such extreme misery. In this condition I was invited to see the patient, with view of an operation. Upon examination the abdomen was immensely distended; uterus crowded completely out of the vagina, legs and feet anasarcus; pulse 100 per minute and weak. Abdomen tender, which tenderness had been of only three weeks standing, indeed, all the more aggravated symptoms had been of recent date; tumor large and apparently movable or disconnected with the peritoneum. With view to relieve the distension and improve respiration, trocar was introduced and about twelve quarts of clear limpid serum drawn off, with effect to relieve somewhat the respiration and make more apparent the size and condition of the tumor. The patient was now not only anxious, but urgently demanded the removal of the tumor, replying to the satement that she was too weak to bear it, "I want to *see how it looks before I die.*" Though this demand of the patient could not justify the operation and should have no determining influence upon the action of the surgeon, still such request, at such time, with plain statement of the danger could not but relieve from responsibility in an operation which afforded at least the only remaining hope. March 5th, 1864, assisted by Dr. Lothrop and private pupils; incision was made down upon the tumor; when exploration, developed extensive attachments, yet so recent as to be easily broken. Trocar was introduced into the body of tumor with view to lessen the size of the mass for its more easy removal; a small quantity of serum escaped without collapse of the walls; upon section the mass was found to be composed of cells surrounded by thickened walls, so dense and unyielding that the size remained undiminished after evacuation of the contents of the cells.

Incision was extended from ensiform cartilage to pubis, and the mass easily raised from its bed. The pedicle by which it was attached was very small, composed mostly of vessels. A strong silk double ligature was passed and tied, a little blood exuded where the puncture was made, and another ligature was placed back of the first, when the pedicle was divided and tumor removed. The abdominal parietes were adjusted and retained by interrupted silk ligatures, the divided pedicle being drawn up and left inclosed within the wound, external to the abdominal cavity.

A tumor which, when emptied of much of its contained fluid, weighed twelve pounds, was thus removed without the loss of any blood at all. The abdominal parieties had been thinned and did not furnish any blood, not twenty drops was lost upon division of the pedicle, so that there was no loss of strength from hæmorrhage. For three days the case progressed favorably, when it became apparent that the system was failing, and she died of exhaustion the fourth day after the operation. The cause of failure was the late period of operation; the woman might and probably would have recovered, could the operation have been made previous to the attack of peritonitis, which so greatly reduced the strength and caused the recent adhesions between the tumor and peritoneum. Though it was attended by unfavorable result, it still appeared almost certain that under any favorable circumstances it would have been successful; indeed it was not the effects of removal, but the delay which proved fatal.

Ovariotomy under favorable circumstances is to be advised, and may be undertaken with hope and confidence. The results of the operation when favorably presented justify this conclusion. Unfavorable cases will occur, but the nature of the disease is such that any considerable proportion of favorable results warrant the procedure even though often attended by fatal effects. The disease is generally sooner or later fatal if left to itself; it is not more than fatal if operated upon, and numerous cases of recovery justify and render imperative the operation, when re-accumulation of fluid after tapping is very rapid, with gradually increasing prostration, rendering it certain that the case must speedily terminate fatally unless relieved. No conscientious surgeon can hesitate under such circumstances; life is only abridged for a very little if unsuccessful, while if the result is favorable a life is saved—a real triumph is achieved. When the respiration is greatly obstructed, and tapping affords little or no relief, extirpation is the only efficient resource; though other plans of relief have been suggested, yet this is really the only resort.

Reliable statistics are as yet wanting to establish the exact ratio of mortality in this operation when performed under proper circumstances. There is little diffiiculty in making the operation, but there is great skill required to know when it is absolutely indicated. One of the greatest embarrassments grows out of the utter impossibility in making correct and positive diagnosis, whatever may be the care in the examination or the experience of the surgeon. Three-tenths of the operations attempted have been abandoned upon discovery of insurmountable obstacles, and in a great many instances no tumor of the ovary has been found. The more solid tumors—fibrous, fleshy or cartalaginous—when discovered attached to the ovary, may be as safely removed, and when productive of similar distresses as the cystic growths, with effusion into abdomen, they as imperatively demand operative interference.

We have said that extirpation was the only relief; perhaps we should, however, say that cases of radical cure, by injection of tincture of iodine have been reported. "Velpeau reports 130 cases treated in this way, of which 64 were cured, 30 died and 36 were temporarily relieved. It is proper to add that it is not certain that the report is reliable."

Others have reported very favorably of the plan of injection, but careful perusal of what has been written will leave the impression that iodine injection is attended by dangers to life scarcely less than extirpation, and that it is seldom followed by permanent relief.

---

*The New Surgeon-General of the United States Armies.*—Dr. Jos. K. Barnes, who for many months past has been Acting Surgeon-General, has been appointed Surgeon-General of the United States Armies, *vice* Dr. Wm. A Hammond, dismissed the service.

